# Analysis of Founder Mutations in Rare Tumors Associated With Hereditary Breast/Ovarian Cancer Reveals a Novel Association of *BRCA2* Mutations with Ampulla of Vater Carcinomas

**DOI:** 10.1371/journal.pone.0161438

**Published:** 2016-08-17

**Authors:** Pedro Pinto, Ana Peixoto, Catarina Santos, Patrícia Rocha, Carla Pinto, Manuela Pinheiro, Luís Leça, Ana Teresa Martins, Verónica Ferreira, Carla Bartosch, Manuel R. Teixeira

**Affiliations:** 1 Department of Genetics, Portuguese Oncology Institute of Porto (IPO-Porto), Porto, Portugal; 2 Department of Pathology, Portuguese Oncology Institute of Porto (IPO-Porto), Porto, Portugal; 3 Institute of Biomedical Sciences Abel Salazar (ICBAS), University of Porto, Porto, Portugal; University of Texas MD Anderson Cancer Center, UNITED STATES

## Abstract

*BRCA1* and *BRCA2* mutations are responsible for hereditary breast and ovarian cancer, but they also confer an increased risk for the development of rarer cancers associated with this syndrome, namely, cancer of the pancreas, male breast, peritoneum, and fallopian tube. The objective of this work was to quantify the contribution of the founder mutations *BRCA2* c.156_157insAlu and *BRCA1* c.3331_3334del for cancer etiology in unselected hospital-based cohorts of Portuguese patients diagnosed with these rarer cancers, by using a strategy that included testing of archival tumor tissue. A total of 102 male breast, 68 pancreatic and 33 peritoneal/fallopian tube carcinoma cases were included in the study. The *BRCA2* c.156_157insAlu mutation was observed with a frequency of 7.8% in male breast cancers, 3.0% in peritoneal/fallopian tube cancers, and 1.6% in pancreatic cancers, with estimated total contributions of germline *BRCA2* mutations of 14.3%, 5.5%, and 2.8%, respectively. No carriers of the *BRCA1* c.3331_3334del mutation were identified. During our study, a patient with an ampulla of Vater carcinoma was incidentally found to carry the *BRCA2* c.156_157insAlu mutation, so we decided to test a consecutive series of additional 15 ampullary carcinomas for *BRCA1/BRCA2* mutations using a combination of direct founder mutation testing and full gene analysis with next generation sequencing. *BRCA2* mutations were observed with a frequency of 14.3% in ampulla of Vater carcinomas. In conclusion, taking into account the implications for both the individuals and their family members, we recommend that patients with these neoplasias should be offered *BRCA1/BRCA2* genetic testing and we here show that it is feasible to test for founder mutations in archival tumor tissue. Furthermore, we identified for the first time a high frequency of germline *BRCA2* mutations in ampullary cancers.

## Introduction

Inherited predisposition to breast cancer is estimated to account for about 5–10% of all cases and is characterized by an autosomal dominant pattern of inheritance, young age at presentation, and association with bilateral breast cancer and ovarian cancer [1, 2]. It has been estimated that up to 1 in 300 and 1 in 800 individuals of the general population carry a *BRCA1* or *BRCA2* mutation, respectively, two genes that are responsible for hereditary breast and ovarian cancer (HBOC). Women carrying germline *BRCA1* mutations have a cumulative risk at 70 years of 60% for breast cancer and 59% for ovarian cancer, whereas *BRCA2* mutations appear to confer a similar risk of breast cancer in females (55%), but a lower risk (17%) for ovarian cancer [3]. Mutation analysis is required to confirm the clinical suspicion of HBOC and to allow appropriate screening and prophylactic measures to carriers in the family [2].

Molecular analyses of the *BRCA1* and *BRCA2* genes have shown that most populations exhibit a wide spectrum of mutations throughout both genes and several founder mutations have been identified in individuals of different ancestries [4]. We have recently characterized the mutational spectrum of the *BRCA1* and *BRCA2* genes in Portuguese HBOC families [5], showing that it is indeed heterogeneous, including two prevalent founder mutations, the *BRCA2* c.156_157insAlu mutation and the *BRCA1* c.3331_3334del mutation. The *BRCA2* c.156_157insAlu mutation was present in 32% of all Portuguese HBOC families and represented 55% of the *BRCA2* mutations, whereas the *BRCA1* c.3331_3334del mutation was present in 11% of all families and 26% of the families with a *BRCA1* mutation, together representing a large proportion of the mutations identified in Portuguese HBOC families. The *BRCA2* c.156_157insAlu mutation has only been reported in families of Portuguese ancestry [5–10], whereas the *BRCA1* c.3331_3334del mutation has been reported in several populations, including Spanish, Canadian and Colombian [11–13].

Mutations in the *BRCA1*/*BRCA2* genes have also been associated with inherited predisposition to other cancers in HBOC families, like those of the prostate, pancreas, male breast, peritoneum, and fallopian tube [14, 15]. We have recently evaluated the contribution of the germline *BRCA1*/*BRCA2* founder mutations for early-onset and/or familial prostate cancer in Portugal [16]. Mutations in *BRCA2* confer a higher risk for developing cancers of the pancreas and male breast, and *BRCA1* mutations seem to be predominantly associated with a higher risk for developing peritoneal and fallopian tube cancer. The objective of this work was to quantify the contribution of the founder mutations *BRCA2* c.156_157insAlu and *BRCA1* c.3331_3334del for cancer etiology in unselected hospital-based cohorts of patients diagnosed with these rarer cancers in Portugal.

## Materials and Methods

### Ethics Statement

This study was approved by the Institutional Ethics Committee of the Portuguese Oncology Institute of Porto (IPO-Porto) (approval number CES 019/08 regarding the use of archival samples for research) and written informed consent was obtained for all patients referred for genetic counselling.

### Subjects

A consecutive series of patients diagnosed at IPO-Porto with any of the cancers strongly associated with HBOC besides female breast, ovarian, and prostate cancer (pancreatic, male breast, peritoneal and fallopian tube) from 1997 to 2013, and from which formalin-fixed, paraffin-embedded (FFPE) tissue was available, was identified. A total of 68 patients with pancreatic tumors (65 ductal adenocarcinomas, 1 mixed ductal-neuroendocrine carcinoma, 1 intraductal papillary mucinous neoplasm with an associated invasive carcinoma and 1 mucinous cystic neoplasm with low grade dysplasia), 27 with male breast invasive ductal carcinomas of no special type and 33 with peritoneal/fallopian tube high-grade serous carcinomas were included in the study with FFPE tissue. Given the large retrospective period of time covered, peritoneal/fallopian tube carcinomas included in the study were limited to those that involved the peritoneum and/or fallopian tube without or only with superficial (<5mm) involvement of the ovary. Furthermore, a consecutive series of 16 patients diagnosed at IPO-Porto with carcinomas of the ampullary region (7 pancreato-biliary type and 9 intestinal type adenocarcinomas), from 1997 to 2013, and from which FFPE tissue was available, were subsequently included. Hematoxylin and eosin-stained slides were carefully reviewed by a pathologist, who delimited tumor and surrounding non-tumoral areas. Family history was not available from any of the patients from whom FFPE tissue was collected. Patients where a mutation was identified during this study were subsequently contacted to provide genetic counselling and to offer their family history.

Additionally, 75 male breast cancer (MBC) patients (39 previously reported by Peixoto et al. [5]) that were referred to the Genetics Department of IPO-Porto for genetic testing of *BRCA1/BRCA2* mutations, not selected for family history of cancer, were also included and peripheral blood samples were collected, giving a total of 102 MBC patients.

### Founder Mutation Screening

**Fig 1:**
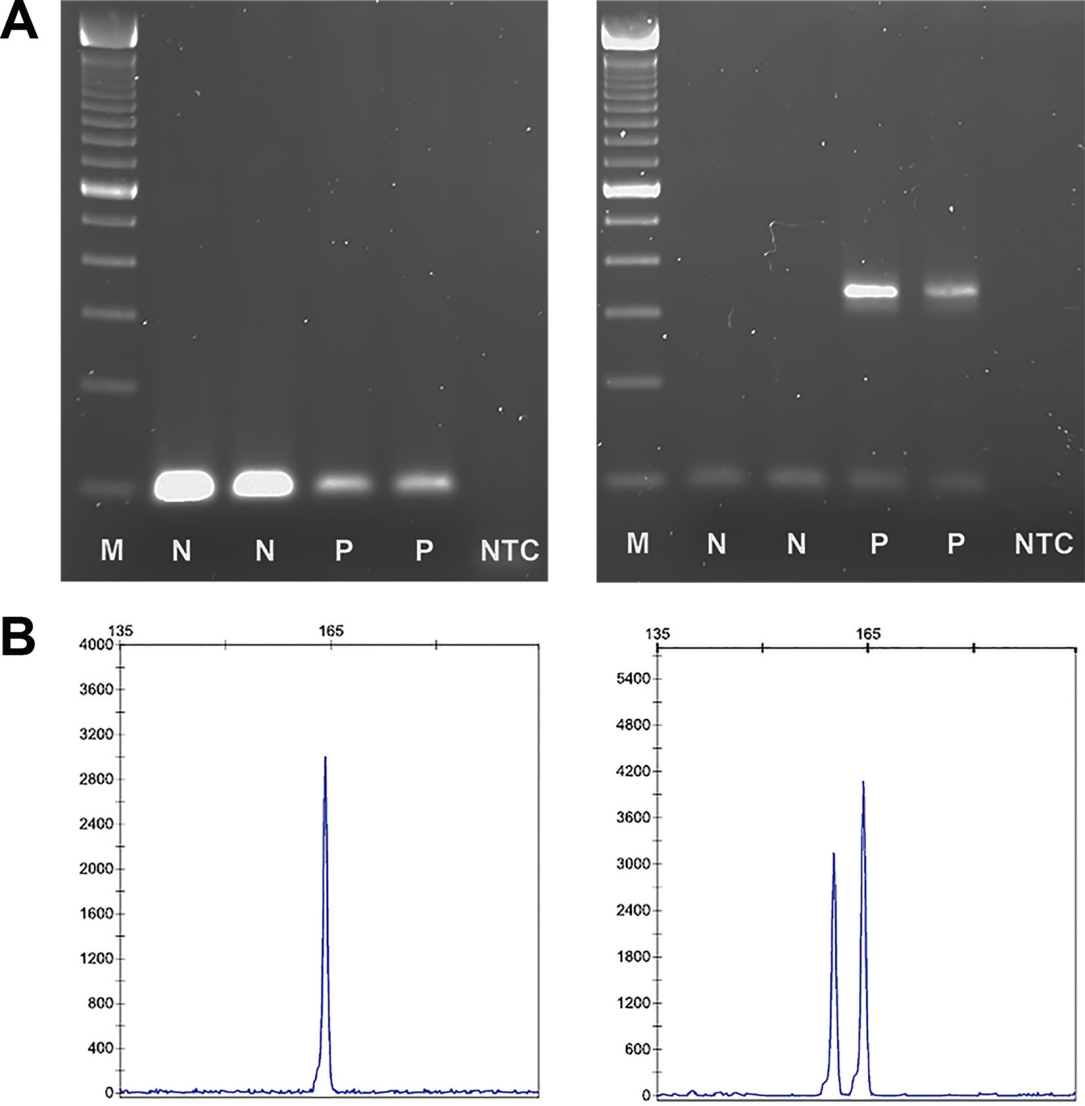
Detection of the *BRCA2* c.156_157insAlu mutation and the *BRCA1* c.3331_3334del mutation in FFPE tissue. (A) Gel electrophoresis pattern of amplification of *BRCA2* exon 3 (left panel) and nested PCR specific for the *BRCA2* c.156_157insAlu mutation (right panel). In non-carriers of the mutation (N) only one amplicon is expected, whereas in carriers (P) a second amplicon is visible in the nested PCR. Non template control (NTC) and 100 bp DNA standard (M) also shown. (B) Capillary electrophoresis pattern from a negative sample (left panel) and a positive control of the *BRCA1* c.3331_3334del mutation (right panel) showing one peak (wild-type alleles) and two peaks (wild-type and mutant allele with 4 bp deletion), respectively.

In FFPE samples, DNA extraction was performed from both tumor and surrounding non-tumoral tissue, whenever available, with the QIAamp DNA FFPE Tissue Kit (QIAGEN, Hilden, Germany) according to the manufacturer’s protocol and DNA quality was evaluated with the NanoDrop ND-1000 (Thermo Fisher Scientific, Waltham, MA). The *BRCA2* c.156_157insAlu mutation was detected by amplification of exon 3 followed by a nested PCR specific for the Alu rearrangement. *BRCA2* exon 3 amplification was performed with the following primers: forward 5`-CTGAACCTGCAGAAGAATCTGAA-3`; reverse 5`-GAAGCCAGCTGATTATAAGATGGTT-3`. The cycling conditions were 94°C for 1 min, 35 cycles of 94°C for 1 min, 52°C for 1 min, and 72°C for 4 min, and a final extension of 72°C for 10 min. In the nested PCR, specific primers for the c.156_157insAlu mutation were used (forward 5`-GACACCATCCCGGCTGAAA-3`; reverse 5`-GAAGCCAGCTGATTATAAGATGGTT-3`) and the cycling conditions were 95°C for 10 min, 25 cycles of 95°C for 45 sec, 62°C for 45 sec, and 72°C for 45 sec, and a final extension of 72°C for 7 min. In the first PCR, due to preferential amplification of the shorter allele, only one amplicon of 111 bp corresponding to the wild-type allele is visible. In the nested PCR, a second amplicon (in positive samples) of about 343 bp corresponding to the allele with the c.156_157insAlu mutation is expected (Fig 1A). Sequence analysis of genomic fragments with the insertion was carried out on an ABI PRISM 310 Genetic Analyser (Applied Biosystems, Carlsbad, CA), using the dye terminator method.

The c.3331_3334del mutation located in *BRCA1* exon 11 was screened using the labelled primers forward 5`-TTAAAGAAGCCAGCTCAAGC-3`and reverse 5`HEX-CTGAAATCAGATATGGAGAG-3`, with the following cycling conditions: 95°C for 10 min, 35 cycles of 95°C for 45 sec, 58°C for 45 sec, and 72°C for 45 sec, and a final extension of 72°C for 10 min. Each sample was run on an ABI PRISM 310 Genetic Analyser together with a fluorescence labeled DNA fragment size standard. The c.3331_3334del mutation status was determined by the presence of one or two peaks corresponding to the wild type and mutated samples, respectively (Fig 1B). All mutations were confirmed by Sanger DNA sequencing.

In patients from whom DNA was extracted from peripheral blood samples, both the *BRCA2* c.156_157insAlu and *BRCA1* c.3331_3334del mutations were screened as previously described [5].

### Next-Generation Sequencing

Next-generation sequencing (NGS) was performed in 12 ampullary tumors in which no founder mutations had been found (in two tumors DNA did not have enough quality). Library preparation was performed using the BRCA Tumor MASTR™ Plus Dx (Multiplicom, Niel, Belgium), which targets the full coding sequence and adjacent intronic regions of the *BRCA1/BRCA2* genes and is optimized for FFPE tissue, following the manufacturer’s protocol. Sequencing was carried out using a standard flow cell in the MiSeq platform (Illumina, Inc., San Diego, CA, USA) in a 2x250 bp paired end run. Sequencing alignment and variant analysis was performed using the software Sophia DDM^®^ version 3.5 (Sophia Genetics, Saint-Sulpice, Switzerland). All variants with an alternative variant frequency ≤5%, minor allele frequency (MAF) >1% and/or intronic variants at more than 12bp away from exon-intron boundaries were excluded. For MAF filtering, data was obtained from the 1000 Genomes Project (1000G; Based on Project Phase III Data), Exome Variant Server (from NHLBI Exome Sequencing Project) and Exome Aggregation Consortium (ExAC) databases.

## Results

**Fig 2:**
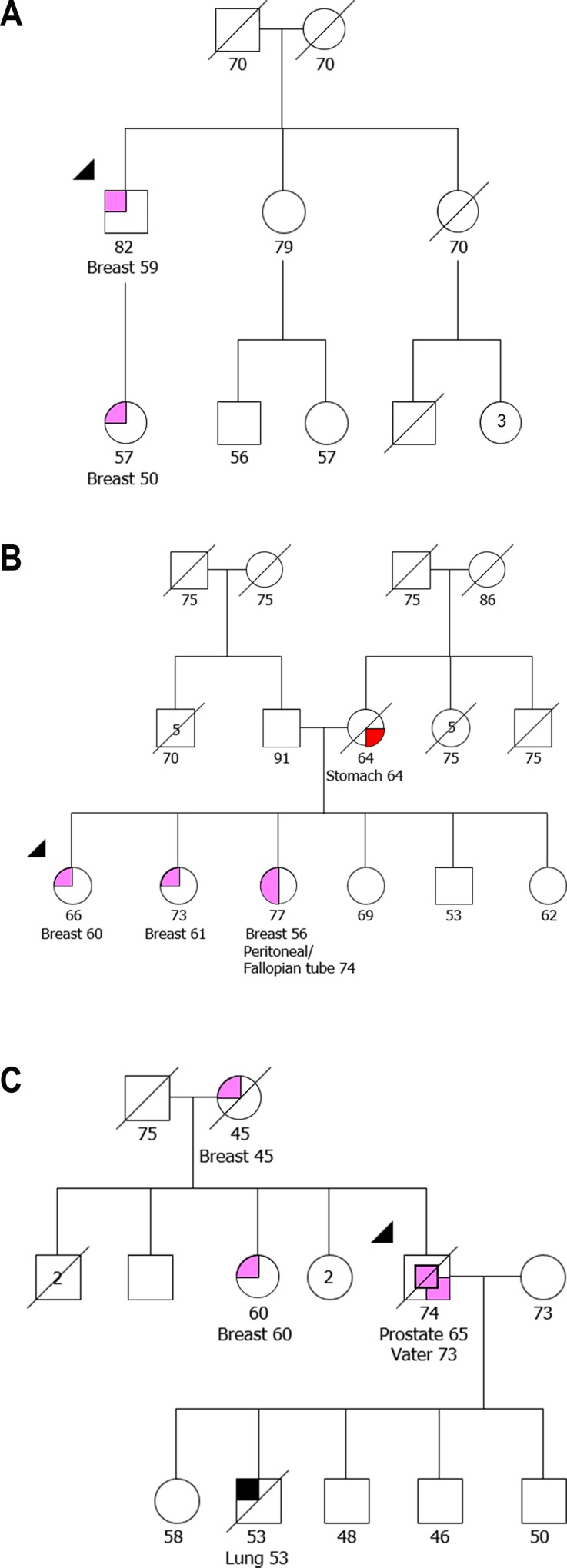
Pedigrees of individuals with the *BRCA2* c.156_157insAlu mutation detected in FFPE tissue. Family of an individual with male breast cancer (A), an individual with peritoneal/fallopian tube cancer (B), and one individual with an ampulla of Vater carcinoma (C). The index case is indicated by an arrow.

A total of 102 MBC patients were analyzed for both the *BRCA2* c.156_157insAlu and *BRCA1* c.3331_3334del mutations. Of the total samples analyzed, eight (7.8%) were positive for the *BRCA2* c.156_157insAlu mutation (three detected in FFPE and five in peripheral blood samples, of which two had previously been reported by us [5]) and the *BRCA1* c.3331_3334del mutation was not identified in any case (Table 1). Of the three patients where the mutation was identified in FFPE tissue, in one the mutation was confirmed to be germline in peripheral blood, another was deceased but belonged to a family that had already been identified in our institution, and in the third it was not possible to test the germline. The age of diagnosis of breast cancer in the *BRCA2* carriers ranged from 47 to 78 years old with a median age of 65 years. It was possible to obtain family history information for seven patients and all of them had a family history of cancers associated with HBOC. One of the patients, besides breast cancer at the age of 47, was also diagnosed with prostate cancer at the age of 55 and four women in his family were diagnosed with breast cancer. Four patients had only family history of female breast cancer, two with one family member (Fig 2A), one with three family members, and the other with five women affected with breast cancer. One patient had three family members affected with female breast cancer and one with ovary cancer. The last patient belongs to a large family with 12 cases of female breast cancer, five cases of prostate cancer and one case with pancreatic cancer.

**Table 1:** Samples analyzed and mutation frequencies observed in tumors associated with HBOC.

Cancer	Samples	*BRCA2* c.156_157insAlu	*BRCA1* c.3331_3334del	% Positive	Estimated *BRCA2* (%)^a^	*BRCA1/BRCA2* (%)
**Male Breast**	102	8	0	7.8	14.3	NA
**Peritoneal / Fallopian Tube**	33	1	0	3.0	5.5	NA
**Pancreatic**	64	1	0	1.6	2.8	NA
**Ampullary**	16	2	0	12.5	NA	14.3^b^

NA–Not available/not applicable

^a^ *BRCA2* c.156_157insAlu represents 55% of the total *BRCA2* mutations identified in Portuguese HBOC families that performed screening of the entire *BRCA1/BRCA2* coding regions [5].

^b^ Frequency of *BRCA1/BRCA2* mutations observed in the 14 samples in which screening of the entire *BRCA1/BRCA2* coding regions was performed.

In the 33 patients with peritoneal/fallopian tube cancer analyzed, none was carrier of the *BRCA1* c.3331_3334del mutation and one patient (3.0%) was a carrier of the *BRCA2* c.156_157insAlu mutation (Table 1). This patient was diagnosed at 74 years old with a high-grade serous carcinoma of the fallopian tube with extensive involvement of the peritoneum. The mutation was confirmed to be germline in peripheral blood and the patient belonged to a family that had already been identified in our institution. She was also diagnosed with breast cancer at 56 years of age and had two sisters with breast cancer (Fig 2B).

An initial series of 69 consecutive cases of putative pancreatic carcinoma was analyzed for the Portuguese founder mutations. Of these, four samples did not have good quality DNA and it was not possible to obtain a result. The *BRCA2* c.156_157insAlu mutation was identified in two samples and no carriers of the *BRCA1* c.3331_3334del mutation were found. When the histopathology material was reviewed it was shown that one of the patients carrying the *BRCA2* c.156_157insAlu mutation had a pancreato-biliary type adenocarcinoma that originated in the ampulla of Vater and not in the pancreas. Hence, a consecutive series of 15 carcinomas of the ampulla of Vater were collected in order to evaluate the contribution of the founder mutations for the pathogenesis of these tumors. One more patient carrying the *BRCA2* c.156_157insAlu mutation was identified in this series, giving a total of two (12.5%) positive samples in the 16 cases of ampullary cancer analyzed for founder mutations (Table 1). The first carrier identified was diagnosed with an adenocarcinoma of the ampulla of Vater at the age of 73 and had been previously diagnosed with prostate cancer at 65 years old. The mutation was confirmed to be germline in peripheral blood and his family history included his mother and one sister diagnosed with breast cancer at the ages of 45 and 60, respectively (Fig 2C). The other patient was diagnosed at 68 years also with a pancreato-biliary type adenocarcinoma of the ampullary region and had no family history of tumors associated with HBOC, only one sister diagnosed with colorectal cancer.

Given the high frequency of the *BRCA2* c.156_157insAlu mutation observed in the ampullary tumors analyzed, we decided to perform screening of the entire *BRCA1/BRCA2* coding regions by NGS. In two of the fourteen negative samples for founder mutations it was not possible to obtain DNA of sufficient quality to perform the analysis. A median coverage of 5100 was obtained for *BRCA1* and of 3770 for *BRCA2* with a minimum coverage of 150 obtained in all samples and only 4.3% of the exons analyzed with a minimum coverage below 500 (data not shown). No additional *BRCA1/BRCA2* deleterious mutations were identified in the 12 samples analyzed by NGS (Table 1).

Of the 64 pancreatic cancer samples where it was possible to obtain a result, one (1.6%) individual carrying the *BRCA2* c.156_157insAlu mutation was identified and none was a carrier of the *BRCA1* c.3331_3334del mutation (Table 1). This patient was diagnosed with an intraductal papillary mucinous neoplasm with an associated invasive carcinoma (ductal adenocarcinoma) of the pancreas at the age of 72 and he had one cousin diagnosed with ovary cancer and another with breast cancer.

## Discussion

The aim of this study was to quantify the contribution of the founder mutations prevalent in Portugal (*BRCA2* c.156_157insAlu and *BRCA1* c.3331_3334del) for cancers associated with HBOC other than the common female breast, ovarian, and prostate cancer, more specifically, the rarer pancreatic, male breast, peritoneal, and fallopian tube cancers. In the 102 MBC patients screened for these mutations, we identified eight (7.8%) carriers of the *BRCA2* c.156_157insAlu mutation. Although these patients were not selected for family history of cancer, all the seven carriers from whom it was possible to obtain information about family history had at least one more family member affected with breast cancer. *BRCA2* mutations are considered the major genetic risk factor for male breast cancer, conferring a lifetime cumulative risk to develop the disease of about 9% [17], but the frequency of these mutations varies considerably between different populations. A study in Southern California detected *BRCA2* mutations in 4% of MBC patients [18], whereas another study in Iceland found mutations in the *BRCA2* gene in 40% of the cases [19]. More recent and larger studies in Israel, Italy and USA described prevalences of 8%, 7%, and 16%, respectively, of *BRCA2* mutations in male breast cancer patients [20–22]. These differences in the frequency of *BRCA2* mutations across different studies can be caused by small sample sizes, mutation screening methods with different sensitivities, mutation screening strategy (entire gene *vs* founder mutations only), presence/absence of family history of tumors associated with HBOC or different classifications of missense mutations. In our study, only the c.156_157insAlu mutation was tested, which accounts for about 55% of all families with pathogenic *BRCA2* mutations in the Portuguese population [5]. Hence, we could expect an overall frequency of about 14.3% of *BRCA2* germline mutations in Portuguese male breast cancer patients in an unselected hospital-based cohort. On the other hand, our data shows that germline *BRCA1* mutations have a limited contribution to the pathogenesis of male breast cancer, which is in accordance with the literature [22, 23].

In the series of 33 peritoneal/fallopian tube cancers analyzed, we identified only one patient (3.0%) carrying the *BRCA2* c.156_157insAlu mutation (estimated total contribution of *BRCA2* mutations of 5.5%) and no carriers of the *BRCA1* c.3331_3334del mutation. There are only a few studies that have analyzed the frequency of *BRCA1/BRCA2* mutations in fallopian tube and peritoneal cancer independently of ovarian cancer. Alsop and colleagues [24] analyzed a series of 152 patients with peritoneal cancer and 40 with fallopian tube cancer and identified a total of 15.8% and 20% patients carrying a *BRCA1/BRCA2* mutation, respectively. Another study performed on 108 patients with fallopian tube cancer identified 21% of patients with a mutation in *BRCA1* and 9% in *BRCA2*, whereas one study performed on 79 patients with peritoneal/fallopian tube cancer identified mutations in *BRCA1/BRCA2* in 23% of the patients [25, 26]. Our low frequency of mutations (3.0%) identified compared to these studies can be explained by the fact that only founder mutations were analyzed and the *BRCA1* founder mutation, which is the gene more commonly associated with these tumors, only represents 11% of all families and 26% of the families identified with a *BRCA1* mutation in Portuguese HBOC families. Whereas our estimation of the contribution of *BRCA2* germline mutations for peritoneal/fallopian tube cancers in hospital-based cohorts is likely to be reliable, the evaluation of the contribution of *BRCA1* mutations may require additional larger studies that include full gene analysis.

We have also evaluated the contribution of *BRCA1/BRCA2* founder mutations in a consecutive series of pancreatic cancers diagnosed at a tertiary cancer center. One of the 64 tumors analyzed (1.6%) had the *BRCA2* c.156_157insAlu mutation. Since this mutation represents 55% of all *BRCA2* germline mutations in our population, it can be estimated that the total contribution of mutations in this gene for pancreatic cancer is about 2.8%. Most of the previous studies conducted for the detection of *BRCA1/BRCA2* mutations in pancreatic cancer were performed in patients with a strong family history of the disease or in individuals with Ashkenazi Jewish ancestry and the reported prevalence of BRCA mutations is variable, ranging from 13% to 19% [27–30]. A recent study was carried out on an unselected, consecutive series of 306 patients from Canada with pancreatic ductal adenocarcinoma and mutations in *BRCA2* were identified in 3.6% of the patients, with a total of 4.6% *BRCA1/BRCA2* carriers identified [31], which does not differ significantly from our estimate for unselected Portuguese patients.

Perhaps the most interesting aspect of our study was the recurrent finding of germline *BRCA2* mutations in carcinomas of the ampullary region. Two of 16 cases of this rare tumor (12.5%) were shown to have the *BRCA2* Portuguese founder mutation, with a 14.3% (2/14) frequency observed when considering only the samples with mutations and those in which all *BRCA1/BRCA2* coding regions were analyzed. Ampullary carcinomas are very rare, accounting for about 0.5% of all gastrointestinal cancers, being often included in the group of pancreato-biliary tumors, but usually have a good prognosis when compared to pancreatic carcinomas [32]. Familial adenomatous polyposis (FAP) patients often develop ampullary adenomas that may progress to ampullary cancer, with a cumulative risk of 10% at the age 60 [33]. Until now, only one study has identified a *BRCA2* mutation in one patient with a carcinoma of the ampulla of Vater, but it was identified in an individual with a family history of breast cancer where this mutation had previously been identified in other family members [34]. To our knowledge, this is the first study that has performed full analysis of the *BRCA1/BRCA2* genes in a consecutive series of ampullary carcinomas. Although the mutation frequency observed is high, our sample size is relatively small and further studies are warranted to confirm the association of *BRCA1/BRCA2* mutations with this rare neoplasia.

The identification of BRCA mutation carriers has implications for both the individuals and their family members, allowing reliable genetic counseling and predictive genetic testing. Female carriers of BRCA mutations can decide whether they want to participate in surveillance protocols and/or perform risk-reducing surgical interventions such as prophylactic bilateral mastectomy and bilateral salpingo-oophorectomy, whereas mutation positive males can engage in breast and/or prostate cancer screening [15]. Moreover, BRCA mutation carriers can also benefit from targeted therapy. BRCA1 and BRCA2 are critical proteins in the process of homologous recombination (HR) repair of double-strand DNA breaks (DSBs). The absence of HR, which is a characteristic of BRCA1/BRCA2 deficient cancer cells, activates error-prone DSB mechanisms like non-homologous end joining (NHEJ) and results in genomic instability [35]. BRCA1/BRCA2-deficient cancers are now recognized as the target for a class of drugs known as PARP (poly (ADP-ribose) polymerase) inhibitors. PARP inhibition, by blocking Base Excision Repair (BER), prevents single-strand break repair and leads to the formation of DSBs, which cannot be accurately repaired in HR-deficient cells and may result in cell death [36]. This synthetic lethality in BRCA-deficient tumors is the basis for the improved response in patients treated with PARP inhibitors [37, 38]. We here show that rarer cancers besides female breast, ovarian, and prostate cancer may be sentinel features that allow the diagnosis of HBOC families and these patients may be included in clinical trials with PARP inhibitors.

In conclusion, we report the contribution of founder mutations to rarer cancers associated with HBOC in Portugal and an optimized method for the detection of these mutations in FFPE tissue (applicable both in neoplastic cells or in the surrounding normal tissue). This optimized method for FFPE tissue is especially important for the detection of the *BRCA2* c.156_157insAlu mutation in patients with Portuguese ancestry, as this prevalent mutation is not readily detectable by standard sequencing technologies [5, 10], therefore allowing its detection even in deceased patients diagnosed with poor prognosis cancers like that of the pancreas. The *BRCA2* c.156_157insAlu mutation was observed with a frequency of 7.8% in male breast cancers, 3.0% in peritoneal/fallopian tube cancers, and 1.6% in pancreatic cancers, with estimated total contributions of germline *BRCA2* mutations of 14.3%, 5.5%, and 2.8%, respectively. In ampullary cancers, we here show for the first time a frequency of 14.3% *BRCA1/BRCA2* mutations after a combination of direct founder mutation testing and full gene analysis in archival tissue with NGS. Taking into account the implications for both the individuals and their family members, we recommend that patients with these neoplasias may be offered *BRCA1/BRCA2* genetic testing and we here show that it is feasible to reliably perform this analysis in FFPE tissue.
